# Influence of Home Composting on Tensile Properties of Commercial Biodegradable Plastic Films

**DOI:** 10.3390/polym13162785

**Published:** 2021-08-19

**Authors:** Maja Rujnić Havstad, Ljerka Juroš, Zvonimir Katančić, Ana Pilipović

**Affiliations:** 1 Faculty of Mechanical Engineering and Naval Architecture, University of Zagreb, 10000 Zagreb, Croatia; ljerka.juros@gmail.com; 2 Faculty of Chemical Engineering and Technology, University of Zagreb, 10000 Zagreb, Croatia; katancic@fkit.hr

**Keywords:** biodegradable polymer, composting, biodegradable film, biodegradable bag, FTIR, garden composting site, poly(butylene adipate-co-butylene terephthalate) (PBAT), TGA, tensile strength, tensile impact strength

## Abstract

In recent years biodegradable plastic films have been increasingly used for various purposes, most often as grocery bags and for collecting bio-waste. Typically, the biodegradation of these films should take place in industrial compost facilities where the biodegradation process occurs under controlled conditions. Nevertheless, many of these films are often disposed of in home composting bins, so the aim of this study was to examine the course of biodegradation of compostable plastic films under uncontrolled conditions in garden composting sites during a period of four months. Mechanical properties were tested on seven different commercially available biodegradable films and bags that were placed in a garden composting bin from February to May. Both tensile properties and tensile-impact strength showed some unexpected results in terms of increase of the properties after the first, second, and third month for some films and bags. The same unpredictability was seen in FTIR and TG analyses.

## 1. Introduction

Plastics are today one of the most used, cheap, and versatile materials, even though they have been introduced a little over a century ago. Products from plastics bring many benefits to society in terms of life quality. One of their biggest advantages is their durability. Unfortunately, it also presents the biggest problem because the rate of degradation does not match their intended service life, so they accumulate in the environment [1].

Over the years, plastic waste has become a major issue, both on land and at sea. Plastic has gone from being hailed as a scientific marvel to being detested as an environmental menace, and one of the symbols of the plastic waste problem is the light grocery store plastic bag [2].

In the beginning of the 21st century, biodegradable plastics attracted public attention as a possible solution to landfill and littering problems, because of their additional end-of-life option biodegradability. Obviously, they will not resolve the littering issue [3], because littering is a social problem that will not be solved by making the material biodegradable, but they have a potential to be biodegraded by biological agents under certain conditions, in a given time. Grocery store bags, and especially the bags for the collection of bio-waste, are one of the most important applications of biodegradable plastic films.

However, since their introduction, there has been a lot of confusion and suspicions about claims of their biodegradability, primarily because plastic products that claimed to be biodegradable did not biodegrade as expected. Biodegradability is an end-of-life option that exploits the power of microorganisms present in the disposal environment to completely remove plastic products designed for biodegradability from the environment via the microbial food time chain in a timely, safe, and efficient manner. The biodegradability of particular plastic material depends on its chemical structure, the types and amount of additive compounds used in their formulation, as well as on the environmental conditions (location and temperature are the most important) under which the product is expected to biodegrade, which is often neglected. Biodegradable plastics are mainly designed to biodegrade under specific conditions—most commonly in industrial composting facilities [4,5]. These sites provide the suitable conditions for microorganisms growth, such as control of moisture (between 50% and 60%), oxygen concentration (over 5%), C/N ratio (in the range 20:1–40:1), and temperature (up to 60 °C), which cannot be regulated in home composting sites [6]. A variety of plastic materials, whether bio-based or fossil-based, are environmentally degradable. A polymer based on a C-C backbone tends to resist degradation, whereas heteroatom-containing polymer backbones provide biodegradability. Therefore, biodegradability can be engineered into polymers by the addition of chemical linkages such as anhydride, ester, or amide bonds, among others. The usual mechanisms of degradation are hydrolysis or enzymatic cleavage of the labile heteroatom bonds, resulting in a scission of the polymer backbone. Microorganisms can eat and digest polymers, as well as initiate a mechanical, chemical, or enzymatic aging [7].

Biodegradation rates of biodegradable plastics are often not as high as predicted from standardized laboratory testing and degradation of end-products under realistic conditions in receiving environments remains largely unknown. Several authors have investigated the compostability of various biodegradable/compostable plastic materials in home composting sites [8,9] and concluded that most of these materials degrade poorly in home composting sites.

Sanchez-Hernandez proposed [10] the use of soil-dwelling and composting earthworms to accelerate the biodegradation rate of biodegradable plastics. Narancic et al. [11] showed that biodegradable plastic blends exhibit excellent biodegradation under industrial composting and even synergy to improve biodegradation in home composting, but they exhibit poor biodegradation and even antagonism in other environments such as aquatic and soil which could lead to long residence times in nature.

The aim of this study was to examine the course of biodegradation of biodegradable plastic films in home composting bin in atmospheric conditions. Typically, the biodegradation of the mentioned films takes place in industrial compost facilities where the biodegradation process occurs under controlled conditions. However, biodegradable films are often disposed of in home, i.e., in garden compost bins, which slows down the biodegradation process. This research was conducted to study biodegradation under uncontrolled conditions on different biodegradable films which are available on the market. Seven different commercially available biodegradable films were placed in a garden composting site in the city of Zagreb from the beginning of February to the beginning of June, with testing taking place every 30 days [12]. Tensile properties and tensile-impact strength were tested on three specimens every 30 days for a period of four months. Degradation of samples was investigated by Fourier-transform infrared spectroscopy and thermogravimetric analysis.

According to the paper of Hazrati et al., the biodegradation kinetics experiment was performed by observing the biomass growth rate over time at different initial substrate concentrations during batch experiments [13]. According to research from authors Tosin et al., the experiment was performed according to the standard ASTM D 5988–12 test method, based on the measurement of CO2 production [14].

However, that is not the case in this paper, as the whole experiment is designed according to the reality of how households dispose of biodegradable bags/films in their composting sites and how long it takes for the plastic biodegradation films to decompose in the home composting bins.

## 2. Materials and Methods

### 2.1. Atmospheric Conditions during Testing

**Figure 1:**
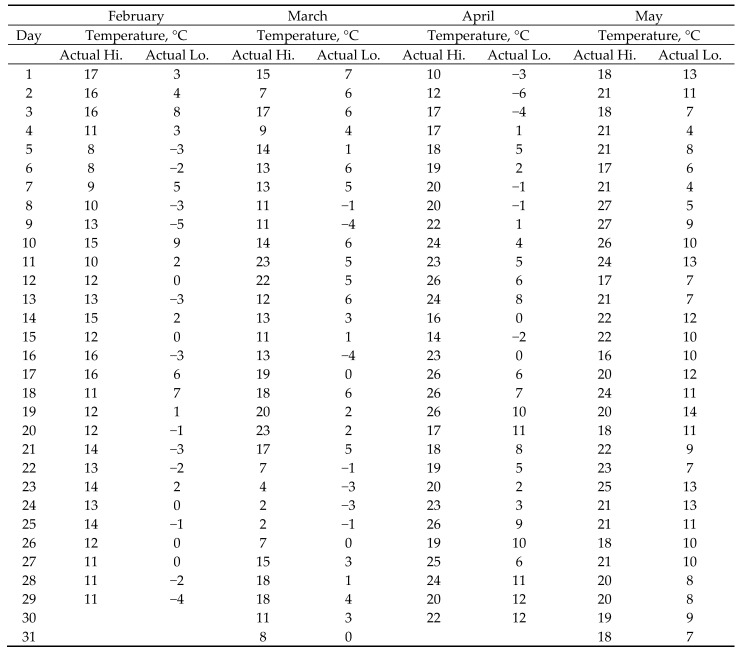
Temperatures in the city of Zagreb from February to the end of May 2020 [16].

The biodegradability property of biodegradable films is defined by various standards, but in a strictly controlled environment of industrial composting plants, where the temperature ranges between 50 and 70 °C and the humidity between 45% and 50% [15]. However, in garden composting sites conditions are significantly different, especially in the winter months. Although the average humidity in the winter and spring months is on average higher than 45–50%, due to the outside temperatures this cannot be equated with the conditions in the composter. The value of relative humidity in the composter is 50%. Temperatures suitable for composting range between 50 and 70 °C, and in this test, they were much lower. February was, as expected, the coldest month, with daytime average temperatures between 6 and 10 °C and nighttime temperatures around −1 °C. March was slightly warmer, with an average daytime temperature between 10 and 12 °C and a nighttime temperature of around 0 °C. In April, daytime temperatures were higher than in March, between 13 and 16 °C, and the average nighttime temperatures ranged between 2 and 6 °C. May, the warmest of these four months, had an average of daytime temperatures between 18 and 22 °C, and nighttime temperatures between 8 and 11 °C (Figure 1). The actual measured temperature values corresponded to the average values shown in the diagram. The composter contains alkaline (basic) soil with an average value of pH 9. The measurement of pH was performed several times during the degradation of the films.

### 2.2. Tested Films and Bags

Seven different biodegradable films, of different thicknesses and collected from different sources, were used for testing (Table 1). Sample 1 (B0) is a bag made of 100% synthetically biodegradable and compostable material from BASF, intended for industrial composting. Sample 2 (SP) is of the same designation as sample B0 but is intended for home composting. Sample 3 (SW) is intended for industrial composting, as well as samples 4 and 5 (labels B1 and B2). Sample 5 is also intended for home composting. Sample 6 (Eco) is a biodegradable plastic film Ecovio F23B1 from BASF, aliphatic copolyester under the trade name Ecoflex with the addition of PLA. The last sample 7 (K) is a biodegradable film produced from 100% corn starch by ALEKO.

The testing of films did not take into account extrusion direction because it was impossible to know whether the specimens were cut in MD (machine direction) or TD (transverse direction), especially after the third and fourth months of decomposition.

### 2.3. Conducting the Experiment

Biodegradable films were placed in house composting bin for a period of 4 months, and after every 30 days the mechanical properties were tested (tensile properties and tensile impact strength). To compare the results, tests were performed before placing in the compost.

**Figure 2:**
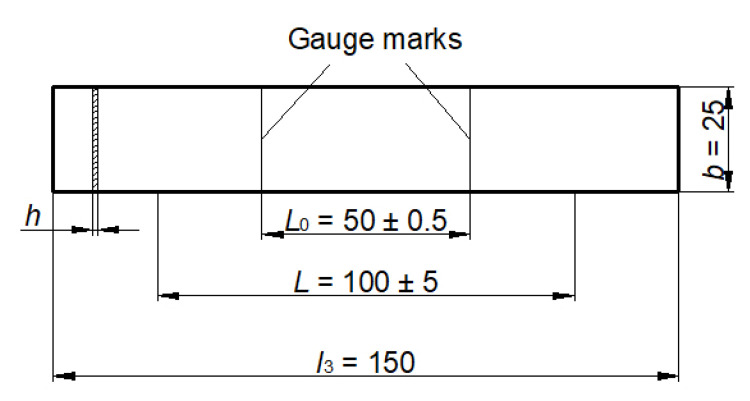
Test specimen for testing tensile properties [17].

Tensile properties were performed according to the standard HRN EN ISO 527-3:2019 on a Shimadzu AGS-X universal testing machine with maximum force of 10 kN and were determined on test specimens of dimensions 25 × 150 mm with thickness less than 1 mm (Figure 2). In the test, the values of tensile strength, strength at break, strain at break and tensile modulus were determined. Three test specimens for each period of composting time (initial film, and after 1 month, 2, 3, and 4 months in composting bin) per film type were examined and the mean and standard deviation were calculated. Tensile tests were performed at a room temperature of 22 °C and at a speed of 50 mm/min.

According to the values of force and elongation, other values are determined according to the equations [17]:

Tensile strength:

(1) $$\sigma_{m=\frac{F_{max}}{A_{0}}},$$

where: *σ_m_* [N/mm^2^]—tensile strength, *F_max_* [N]—max. force, *A*_0_ [mm^2^]—initial cross section.

Strength at break:

(2) $$\sigma_{b=\frac{F_{b}}{A_{0}}},$$

where: *σ_b_* [N/mm^2^]—breaking strength, *F_b_* [N]—breaking force, *A*_0_ [mm^2^]—initial cross section.

Strain at break:

(3) $$\varepsilon_{b=\;\frac{\Delta l}{l_{0}}*100\%},$$

where: *ε_b_* [%]—strain at break, Δ*l* [mm]—increase of the specimen length, *l*_0_ [mm]—gauge length of test specimen.

Tensile modulus:

(4) $$E=\frac{\sigma }{\varepsilon },$$

where: *E* [N/mm^2^]—tensile modulus, *σ* [N/mm^2^]—stress, *ε* [%]—strain.

**Figure 3:**
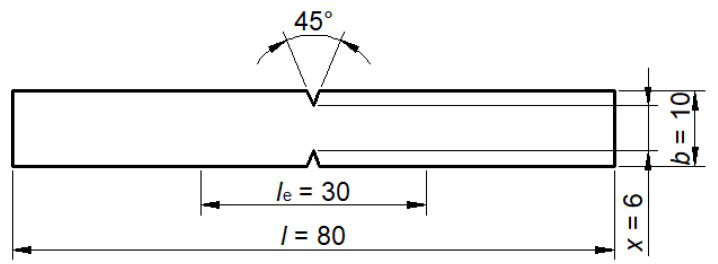
Test specimen for testing tensile-impact strength [18].

Furthermore, the tensile-impact strength according to the standard HRN EN ISO 8256:2004 was tested by the method A, which determines the strength for test specimens of dimensions 80 × 10/6 mm (*l* × *b*/*x*) with a thickness of less than 4 mm (Figure 3). The tests were performed at room temperature of 23 °C.

According to the method A, crosshead mass is 30 ± 1 g for the pendulum 2 J.

To determine the tensile-impact strength, it is necessary to calculate the energy correction and then calculate the tensile-impact strength according to the following equations [18]:

Correction *E_q_* due to the plastic deformation and kinetic energy of the crosshead:

(5) $$E_{q}=\frac{E_{max}\cdot \mu \cdot \left(3+\mu \right)}{2\cdot \left(1+\mu \right)},$$

where: *E_q_* [J]—energy correction due to the plastic deformation and kinetic energy of the crosshead, *E_max_* = 0.5 J [J]—maximum impact energy of the pendulum, *μ*—mass of the crosshead (*m_cr_* = 0.0277 kg) divided by the reduced mass of the pendulum (*m_cr_*/*m_p_*).

Reduced mass *m_p_* of the pendulum:

(6) $$m_{p}=\frac{E_{max}}{g\cdot L_{p}\cdot \left(1-cos\alpha \right)},$$

where: *g* = 9.80655 [m/s^2^]—acceleration due to gravity, *L_p_* = 0.2 [m]—pendulum length, *α* = 160° = 2.79252 rad—angle between the position of the pendulum at its maximum and minimum height.

Energy correction for method A:

(7) $$E_{c}=E_{s}-E_{q},$$

where: *E_c_* [J]—corrected tensile-impact energy, *E_s_* [J]—impact energy absorbed during the impact.

Tensile-impact strength:

(8) $$a_{tN}=\frac{E_{c}}{x\cdot h}\cdot 10^{3},$$

where: *a_tN_* [kJ/m^2^]—notched tensile-impact strength, *x* [mm]—distance between the notches, *h* [mm]—thickness.

Five types of biodegradable films were also characterized by Fourier transform infrared spectroscopy (FTIR). Perkin Elmer Spectrum One spectrometer was used, in the range from 4000 to 650 cm^−1^ with the resolution of 4 cm^−1^. Spectra were collected in reflectance mode using ATR chamber equipped with ZnSe crystal. Samples were wiped before measurement with ethanol to remove dust or other surface impurities. The effect of degradation on the spectra was evaluated using FT-IR and TGA after 3 months, because some films had completely decomposed after 4 months.

Thermogravimetric analysis (TGA) of samples was carried out using a TA Instruments Q500 analyzer. The results are obtained in temperature range from room temperature to 600 °C, at heating rate of 10 °C/min under the N_2_ atmosphere with a flow rate of 60 mL/min during analysis. Weight of each sample was approximately 3 mg. Three replicates were run for each sample and the average value was reported. Uncertainty of initial mass loss and maximum loss rate temperatures was less than 3.1 °C while char residue uncertainty was 1.6 mass % (2σ).

## 3. Results

**Figure 4:**
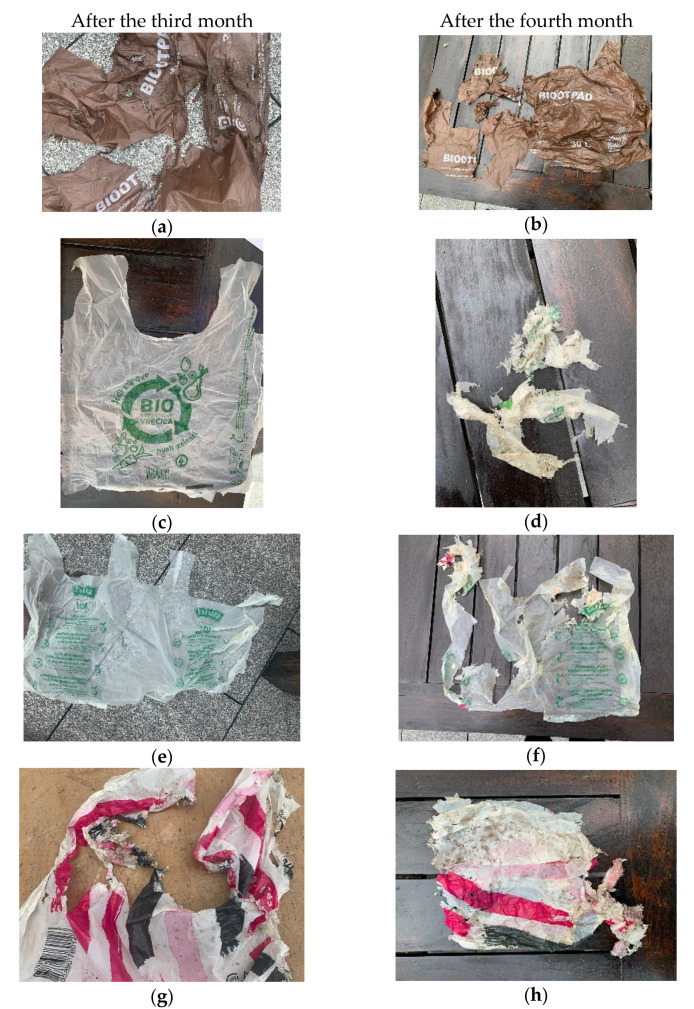

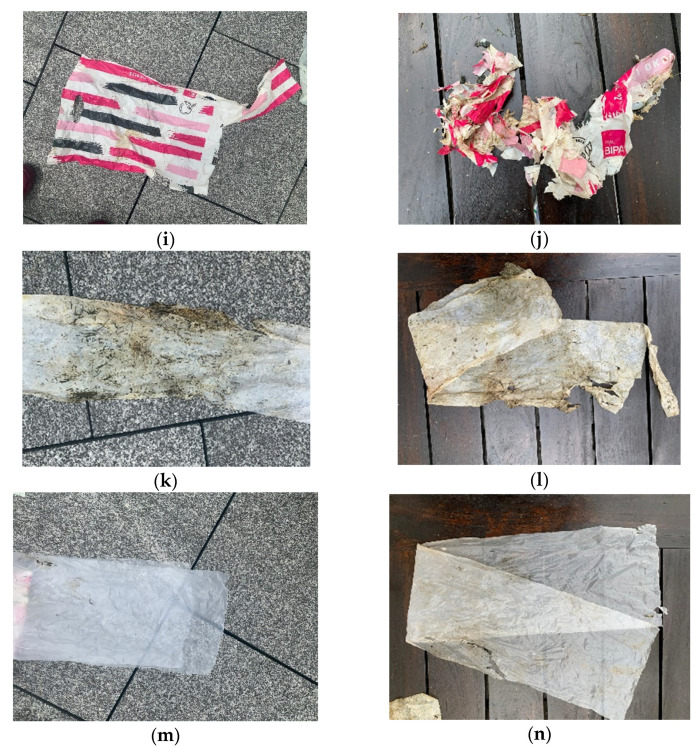
Degradation of films after the third and fourth month: (**a**) B0 after 3 months, (**b**) B0 after 4 months, (**c**) SP after 3 months, (**d**) SP after 4 months, (**e**) SW after 3 months, (**f**) SW after 4 months, (**g**) B1 after 3 months, (**h**) B1 after 4 months, (**i**) B2 after 3 months, (**j**) B2 after 4 months, (**k**) Eco after 3 months, (**l**) Eco after 4 months, (**m**) K after 3 months, (**n**) K after 4 months. The figure shows all 7 films only after the third and the fourth month because in most cases after the first and even after the second month no change and decomposition was visually observed.

The testing started in February 2020. The compost contained mostly garden waste, but due to low outdoor temperatures, no biodegradation was observed during the first month. After the second month, the beginning of decomposition was observed, but more significant decomposition was observed only after third and fourth month, when the temperatures increased according to Figure 1. Furthermore, wet bio-waste was added to the compost. After four months, some of the test samples degraded so much that test specimens could not be prepared (Figure 4).

### 3.1. Tensile Properties

Table 2, Table 3, Table 4 and Table 5 show the results of tensile properties testing over a period of four months. The tests were performed on three test specimens, and the table shows the average values and standard deviations. Tensile modulus was measured at the values of stress 1 and 3 N/mm^2^.

**Figure 5:**
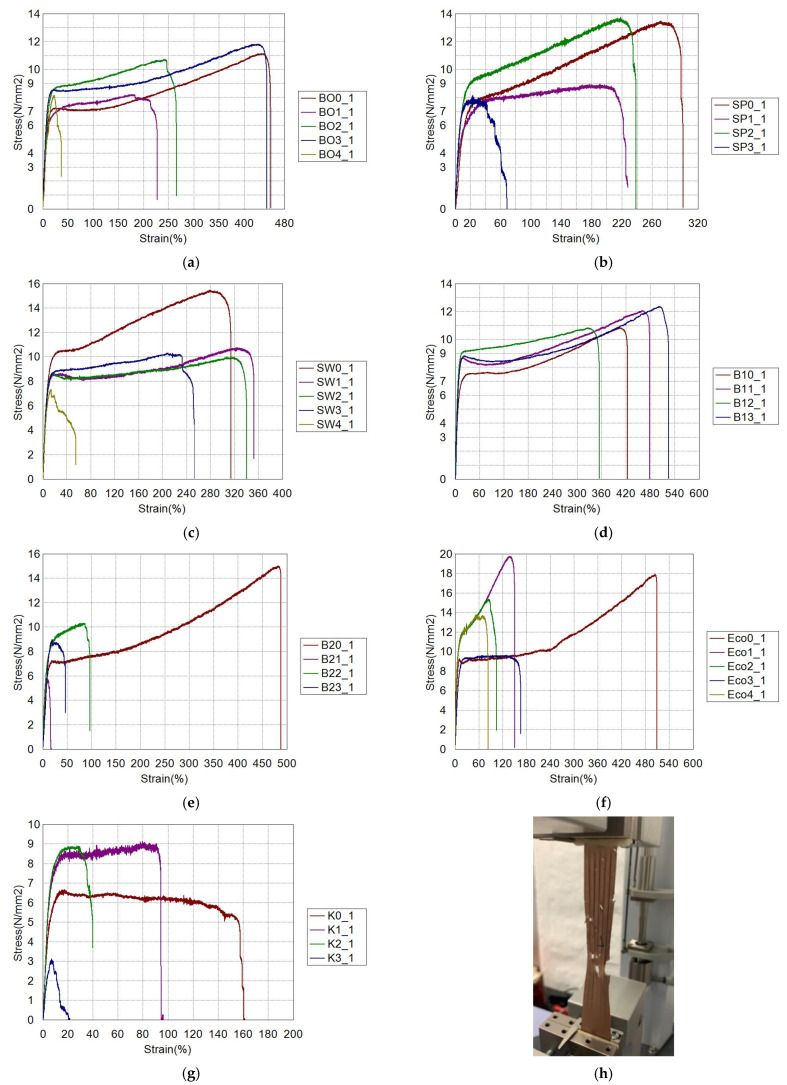
Testing procedure and tensile stress-strain diagrams for groups of film samples in the period of 0, 1, 2, 3, and 4 months: (**a**) B0, (**b**) SP, (**c**) SW, (**d**) B1, (**e**) B2, (**f**) ECO, (**g**) K, (**h**) procedures of testing. The diagrams show that only after 3 or 4 months of decomposition a greater decrease in mechanical properties is observed.

Figure 5 shows the tensile stress-strain diagrams for individual films during all four months. Only the mean curves are shown in the diagrams.

### 3.2. Tensile-Impact Strength

**Figure 6:**
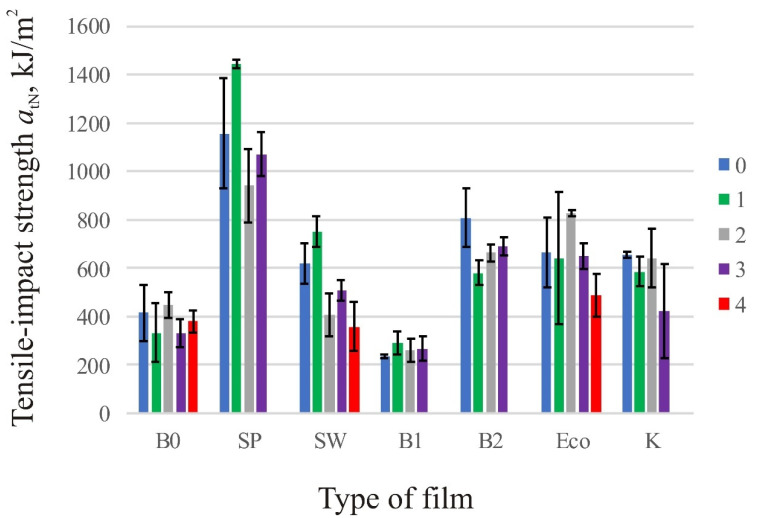
Tensile-impact strength of the film samples in the period of 0, 1, 2, 3, and 4 months. The figure also shows the error bars through the standard deviation.

To calculate tensile-impact strength using correction energies according to Equations (5) to (8), data of reduced mass of the pendulum *m_p_* = 0.1314 kg and mass *μ* = 0.21076 kg were determined. Table 6 and Figure 6 show the tensile-impact strength for individual films during the whole decomposition period. Only the mean curves are shown in Figure 6.

### 3.3. Fourier-Transform Infrared Spectroscopy

**Figure 7:**
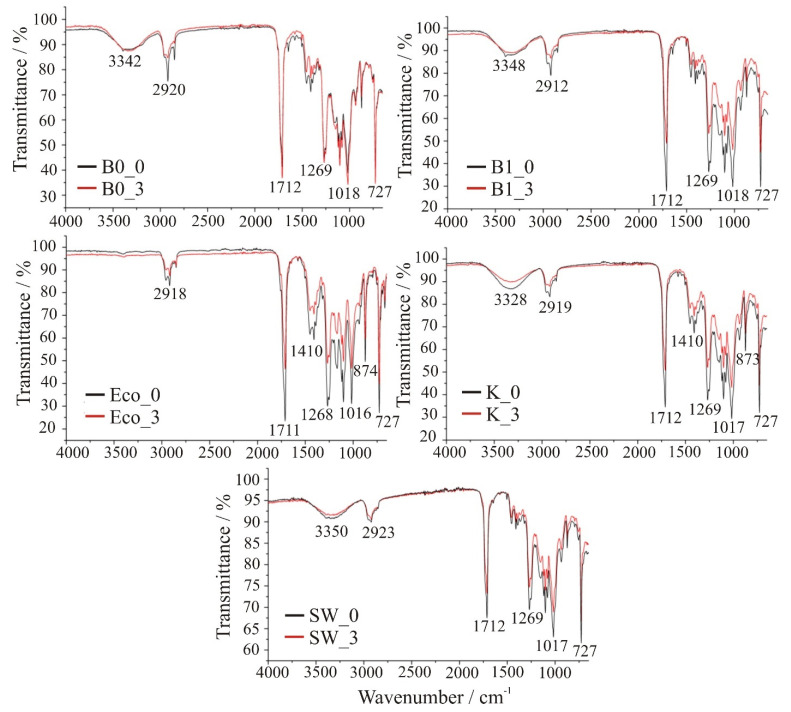
FT-IR spectra of tested samples before and after 3 months of decomposition.

Figure 7 shows FT-IR spectra for individual films before testing and after three months.

### 3.4. Thermogravimetric Analysis

**Figure 8:**
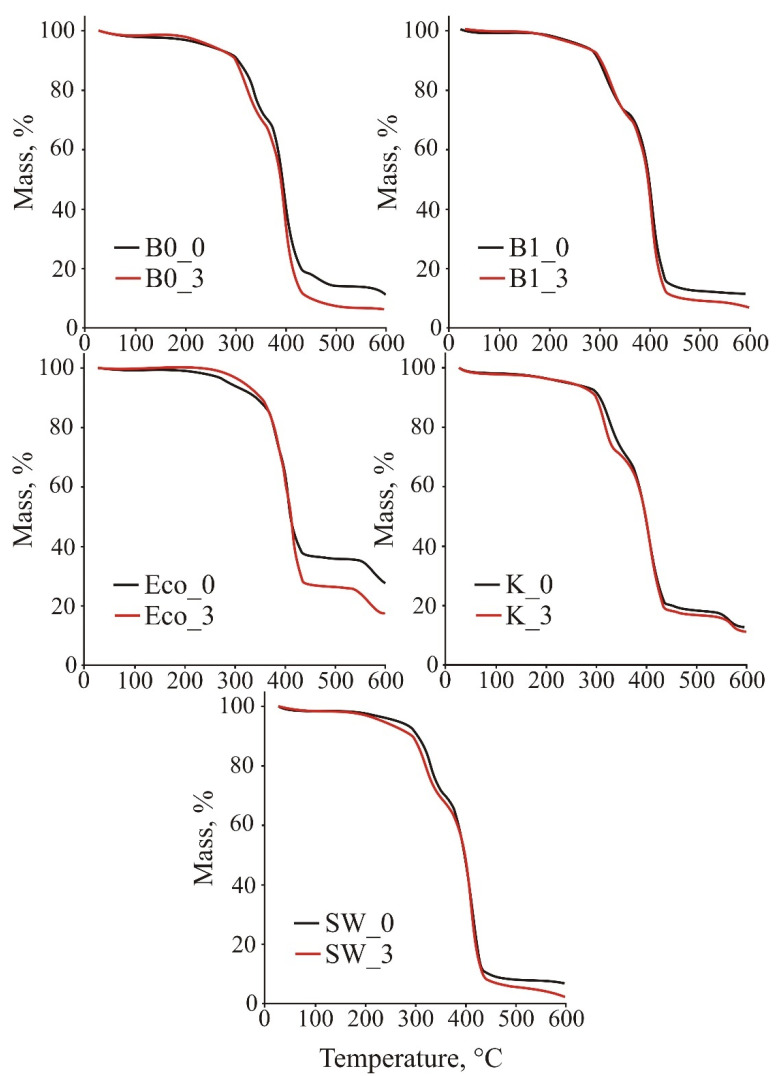
TG curves of tested samples before and after 3 months of decomposition.

Table 7 and Figure 8 shows the TGA results for individual films before degradation and after three months.

## 4. Discussion

The films marked with BO, SP, SW, K, and B2 have in common that the last measurements (after the third or fourth month) are the lowest values of all measurements, which is to be expected. What was not expected was an increase in the values of strain at break and tensile strength after initial test (0). For Eco and B1 foils the last results of strain at break and tensile strength are not the lowest of all, and for film B1 the test 3 gave the highest values. Unusually, film B1 was biodegraded and was not tested in the fourth month, but only 30 days, earlier the values of the aforementioned properties were the highest.

Loss in tensile properties is the most relevant practical criterion to determine degradation of biodegradable films. Czaja-Jagielska studied tensile properties of potato starch compostable films, and reported the decrease in tensile strength with increasing time of composting [19]. Mostafa studied mechanical properties of various biodegradable films under different soil types. The tensile strength of almost all films tested showed a lag phase and no significant decrease until the third month [20].

Scarascia-Mugnozza studied mechanical properties decay of biodegradable films for agricultural mulching in real scale experiment, and found out that properties at break (elongation at break and stress at break) are more significant for the evaluation of the effect of degradation in such highly deformable materials as the mulching films. Properties were tested periodically in the period of 124 days, and there was a sudden increase of above mentioned properties after two months on the out-soil portions of degradable mulch. The increase did not happen for the in-soil films [21].

The results of tensile-impact strength supported the results of tensile properties. For all the films, the values tensile-impact strength did not decrease continuously, but oscillated. For bags and films marked with SW, K, and ECO, the last value of tensile-impact strength is also the lowest, while for the other films this is not the case. For some films the test value after the second month was the highest, while for the others it was the lowest and it is not possible to determine a pattern of behavior that would apply to all the films.

The films labelled SP and B2 are the only ones marked as suitable for home composting, which proved to be correct, because neither bag could be tested after four months due to excessive decomposition. This conclusion was reached by the authors of Adamcová et al. in their studies where degradable bags/films degraded completely after 12 weeks (three months). However, in their research, the testing was conducted in a laboratory, while in a real environment such as ours testing, it still took a month longer [1]. Films that have decomposed too much for test 4 and were not designated for home composting are SW, K, and B2 films, which was probably the result of the weather conditions. The films were placed in the composter in February, when the outside temperatures were low, and biodegradation was expected to be slower during that period than in May, when most of the changes became visible.

There is no tendency to explain the behavior of the material, i.e., the reason for the increase of tensile properties and tensile-impact strength after a few months of degrading in home composting bins. However, as extrusion direction was not taken into consideration and all the films exhibit different properties in MD and TD, it is possible that some increase of tensile properties can be contributed to testing of film in different extrusion directions.

FT-IR spectra of samples B0, B1, ECO, K, and SW, before and after three months of decomposition are shown in Figure 6. Since all the films were commercially available, their true chemical composition was not known, so FT-IR was also used to identify the material. It can be seen that all spectra are similar with the sample ECO showing slightly different spectra in the region around 3340 cm^−1^. From the comparison with literature [22,23], it can be concluded that all films show typical vibrations of poly(butylene adipate-co-butylene terephthalate) (PBAT). The peaks at ~2920 and 2850 cm^−1^ correspond to the asymmetric and symmetric stretching of the aliphatic C–H groups, respectively. The sharp peaks at 1712 and 727 cm^−1^ can be attributed to the C=O stretching vibration of the ester group and the out-of-plane C–H deformation of the aromatic ring, respectively. The bands corresponding to the C‒O and C-O-C stretching vibrations are identified at ~1269 and ~1100 cm^−1^, respectively, while the absorption bands at 1018 and at 1410 cm^−1^ are characteristic group stretchings of the phenylene group [24]. PBAT belongs to the group of biodegradable polyesters, so its application for biodegradable films seems reasonable. The difference between ECO and the other samples is that ECO has only vibrations typical of PBAT, while all the others have a broad peak centered at ~3340 cm^−1^. Such peaks are usually attributed to OH groups, which are not found in PBAT. A possible explanation is that the films, except ECO, are polymer blends of PBAT and thermoplastic starch (TPS), which has -OH groups in its structure. Other vibrations in TPS (C–H, C=O, C–O, C–C) are common with PBAT, so it is not possible to distinguish them [25].

Comparison of the spectra before and after three months of decomposition shows that all samples have identical peaks, but with lower intensities, indicating that the chemical composition has not changed. The vibrational intensities of the chemical groups present decrease randomly, suggesting that decomposition occurs randomly at different locations in the chains.

Thermogravimetric curves of the samples before and after three months of decomposition and typical values obtained from these curves are shown in Figure 7 and Table 6. The thermal stability of all samples, except for the sample ECO, is similar. The degradation occurs in two distinct steps, the first between 250 and 350 °C, the second between 350 and 450 °C. The FT -IR results indicate that the films are blends of PBAT and TPS, so the first step can be attributed to the degradation of the –OH groups in the starch [26], while the second step corresponds to the degradation of PBAT. The approximate percentage of TPS in the films, evaluated from the mass loss in the first degradation step, is between 20% and 25%. The sample ECO was shown by FT-IR to have a different chemical composition, i.e., it consists only of PBAT, and therefore the thermal behavior was different. Although it also has two degradation steps, the first one is very small around 300 °C, so it is possible that it has only a small fraction of TPS, which was not detectable with FT-IR. Almost all of the degradation occurs between 350 and 450 °C, where PBAT is degraded. This sample also had the highest residual mass after pyrolysis, where 28% of the mass remained, while this value ranged from 7.0% to 12.8% for the other samples.

When comparing the TGA results of fresh films and after three months of decomposition, the only clear trend is the residual mass, which decreases by 10% to 60% after decomposition. The onset of decomposition (*T*_95_) varies, in some cases the decomposed samples show an increase in the onset of decomposition (B0, ECO), while some start to decompose earlier (B1, SW). Maximum degradation rate (*T*_max_) temperatures are either about the same for the decomposed samples or even shifted by about 5 °C to higher temperatures.

## 5. Conclusions

In this paper, mechanical properties were tested on seven different commercially available biodegradable films and bags that were placed in a garden composting bin from February to May. Tensile properties, such as tensile strength and strain at break, showed unexpected results in terms of increase of the properties after the first, second, and third month for some films and bags. Tensile-impact strength tests also showed results that were not predicted, and these are increases and decreases over months according to an irregular pattern. Each bag and film showed some specific behavior in terms of mechanical properties, so it is not possible to generalize and draw final conclusions. The same unpredictability was seen in FT-IR and TG analyses. Although FT-IR showed decreased intensities of vibrations suggesting degradation, the same was not clearly visible in TGA. Also, the tested bags and films were of different thicknesses and purposes according to the type of composting, so it is difficult to compare them. One of the influencing factors, high temperature, was left out most of the time and the results would certainly have been different if the same experiment had been conducted in the summer months.

The bags and films that possessed the label of home compostability indeed mostly decomposed in the home composting bin, but not completely. Even some bags bearing the label of being compostable only in industrial composting sites have mostly fragmented. In order to have a clearer insight into the behavior of different bags and films during the period in the composting bin, further analysis and testing of some other physical properties is needed.

## Figures and Tables

**Table 1:** Tested biodegradable films.

Label	Certificates	Average Thickness, mm	Photo
B0	DIN CERTCO 7P0324 TÜV AUSTRIA S0426 Industrial composting	0.028	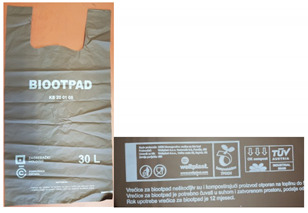
SP	DIN CERTCO 7P03240 TÜV AUSTRIA S0426 Home composting	0.011	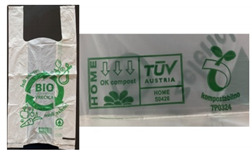
SW	DIN CERTCO 9G0087 TÜV AUSTRIA 7P0002 Industrial composting	0.022	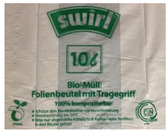
B1, B2	DIN CERTCO 7P0324 TÜV AUSTRIA S0426 B1 − Industrial composting B2 − Industrial and home composting	B1−0.04 B2−0.018	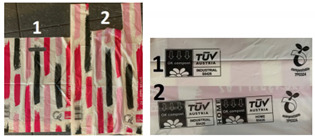
Eco	DIN CERTCO 7W0188 Industrial composting	0.017	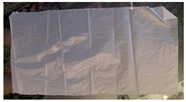
K	-	0.019	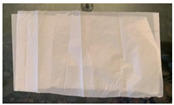

**Table 2:** Tensile strength.

	Tensile Strength, MPa				
Sample Name/ Months	0	1	2	3	4
B0	11.2 ± 0.5	8.7 ± 0.9	10.9 ± 0.9	12.1 ± 1.9	8.3 ± 0.1
SP	13.7 ± 2.2	9.9 ± 0.8	14.4 ± 4.3	8.4 ± 0.6	-
SW	15.9 ± 2.0	11.5 ± 1.7	10.2 ± 0.9	10.7 ± 0.8	7.6 ± 1.7
B1	10.9 ± 1.7	12.2 ± 1.6	10.9 ± 0.8	12.5 ± 1.7	-
B2	15.3 ± 0.9	6.2 ± 2.4	10.5 ± 0.9	9.3 ± 1.4	-
Eco	18.2 ± 3.6	20.2 ± 4.4	16.1 ± 6.2	10.5 ± 1.2	14.7 ± 3.5
K	7.3 ± 0.3	9.4 ± 0.8	9.0 ± 0.4	3.8 ± 1.3	-

**Table 3:** Tensile strength at break.

	Tensile Strength at Break, MPa				
Sample Name/ Months	0	1	2	3	4
B0	11.1 ± 0.6	5.4 ± 3	7.9 ± 4.3	9.5 ± 3.1	4.8 ± 1.4
SP	10.0 ± 2.9	4.6 ± 1.3	10.9 ± 5.9	2.4 ± 1.0	-
SW	12.8 ± 5.1	8.3 ± 3	7.3 ± 1.8	6.7 ± 3.2	3.2 ± 3.1
B1	9.9 ± 1.7	10.7 ± 1.2	7.7 ± 1.5	9.5 ± 3.5	-
B2	14.3 ± 1.4	3.4 ± 2.4	8.2 ± 3.2	5.5 ± 2.3	-
Eco	17 ± 3.9	16.4 ± 6.2	10.9 ± 7.5	6.8 ± 2.1	10 ± 1.3
K	4.1 ± 0.6	7.8 ± 1.0	4.7 ± 1.0	0.4 ± 0.05	-

**Table 4:** Tensile strain at break.

	Tensile Strain at Break, %				
Sample Name/ Months	0	1	2	3	4
B0	441 ± 10.1	227.3 ± 142.5	265.5 ± 56.9	444.9 ± 83.4	36.8 ± 7.7
SP	299 ± 65.6	225 ± 126.6	237.4 ± 80.2	66.7 ± 27.9	-
SW	313.6 ± 73.1	352.4 ± 162.2	339.2 ± 27.0	252 ± 111.7	55.5 ± 33.2
B1	423.3 ± 156.2	477.8 ± 75.7	355 ± 53.7	525.2 ± 100.6	-
B2	487.7 ± 45.0	15.8 ± 6.7	96.1 ± 40.8	47 ± 14.9	-
Eco	508.8 ± 24.0	150.6 ± 28.6	101.7 ± 46.5	165.5 ± 124.6	83.1 ± 43.0
K	159.5 ± 90.9	104 ± 21.6	40.1 ± 13.9	18.1 ± 13.5	-

**Table 5:** Tensile modulus.

	Tensile Modulus, MPa				
Sample Name/ Months	0	1	2	3	4
B0	98.1 ± 24.5	103.7 ± 17.3	91.8 ± 16.2	119.4 ± 20.8	103.4 ± 5.8
SP	73.9 ± 11.8	102.3 ± 2.3	105.5 ± 14.3	113.1 ± 27.9	-
SW	151.3 ± 28.8	123.7 ± 26.5	133.6 ± 27.9	144.5 ± 10.1	116.3 ± 17.1
B1	105.2 ± 14.5	128.3 ± 19.2	122.4 ± 19.9	130.6 ± 10.0	-
B2	100.5 ± 11.1	119.2 ± 21.5	118.1 ± 25.4	121.1 ± 48.7	-
Eco	182.3 ± 41.0	199.2 ± 55.3	243.7 ± 36.2	153 ± 40.7	230 ± 56.0
K	153.7 ± 0.6	151.8 ± 8.7	176.2 ± 23.8	106.6 ± 15.8	-

**Table 6:** Tensile-impact strength.

	Tensile-Impact Strength, kJ/m^2^				
Sample Name/ Months	0	1	2	3	4
B0	414.4 ± 117.6	331.7 ± 121.2	448.6 ± 53.4	329.5 ± 57.2	379.5 ± 46.5
SP	1156.8 ± 226.1	1444.2 ± 16.5	940.5 ± 149.7	1070.8 ± 89.4	-
SW	619.1 ± 83.0	749.9 ± 63.2	406.6 ± 89.6	506.4 ± 43.9	358 ± 102.1
B1	234.1 ± 8.1	292.1 ± 48.6	257.8 ± 48.2	266 ± 50.3	-
B2	807 ± 121.1	578.9 ± 50.7	662.9 ± 33.8	690.2 ± 38.1	-
Eco	664 ± 143.6	640.9 ± 273.6	826 ± 14.1	651.5 ± 53.0	487 ± 88.9
K	654.8 ± 12.9	584.7 ± 61.5	641.7 ± 120.6	421.8 ± 194.2	-

**Table 7:** TGA results of studied films; onset degradation temperature (*T*_95_), temperature of maximum degradation rate (*T*_max_), and the char residue at 600 °C (*r*_600_).

	*T*_95_ (°C)	*T*_max_ (°C)	*r*_600_ (%)
B0_0	238.8	390.3	11.4
B0_3	252.7	395.2	6.2
B1_0	261.4	411.0	11.0
B1_3	253.2	411.3	6.9
Eco_0	293.2	401.6	28.0
Eco_3	318.9	405.2	17.9
K_0	260.5	410.3	12.8
K_3	259.1	410.4	11.3
SW_0	260.3	408.6	7.0
SW_3	234.3	413.9	2.5

## Data Availability

Publicly available datasets were analyzed in this study. This data can be found here: https://repozitorij.fsb.unizg.hr/islandora/object/fsb%3A6094.
